# Persistent Notochord in a Fetus with COL2A1 Mutation

**DOI:** 10.1155/2015/935204

**Published:** 2015-09-08

**Authors:** Elisabeth Codsi, Brian C. Brost, Arij Faksh, Amber K. Volk, Kristi S. Borowski

**Affiliations:** ^1^Mayo Clinic, 200 First Street SW, Rochester, MN 55905, USA; ^2^Wake Forest School of Medicine, 1 Medical Center Boulevard, Winston Salem, NC 2715, USA; ^3^NxGen MDx, 801 Broadway Avenue NW, Suite 203, Grand Rapids, MI 49504, USA

## Abstract

Multiple anomalies including micromelia, poor mineralization of the vertebrae, and a persistent notochord were identified on second trimester ultrasound in a fetus with a *COL2A1* mutation. To our knowledge, this represents the first case of a persistent notochord associated with a *COL2A1* mutation in humans. In this case report, we describe ultrasound and postmortem findings and review the pathogenesis associated with a persistent notochord.

## 1. Introduction

The notochord is an embryonic axial skeletal component found in all chordates. As the vertebral bodies form, this transient structure transforms into the nucleus pulposus of the intervertebral discs [1]. Here we report the case of a persistent notochordal canal associated with a COL2A1 mutation and review the ultrasound findings and pathogenesis of a persistent notochord.

## 2. Case Report

**Figure 1:**
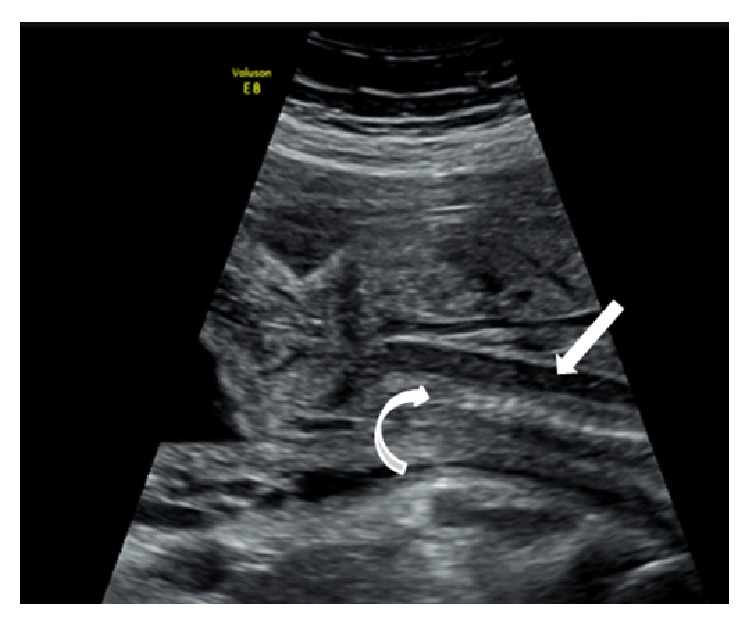
Sagittal view of the fetus showing hypomineralization of the vertebrae (curved arrow) and an echolucent and tubular structure anterior to the spine (straight arrow), ending at the end of the sacrum.

**Figure 2:**
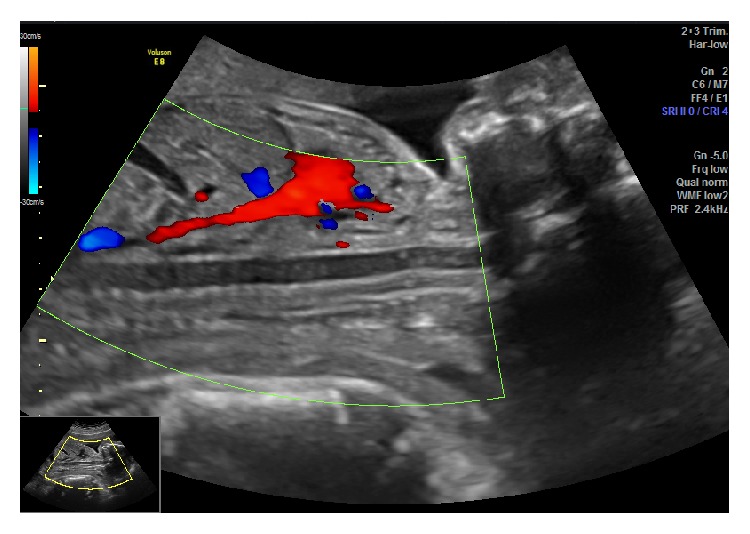
Color Doppler showing absence of flow through the notochord.

A 30-year-old gravida 3 para 1 was referred to our center at 23 weeks of gestation based on last menstrual period for a possible fetal skeletal dysplasia. The patient's past obstetrical history was relevant for a term vaginal delivery and a first trimester miscarriage at approximately 6 weeks of gestation. There was no family history of congenital anomalies or genetic syndromes. Routine second trimester ultrasound performed at her local hospital showed an abnormal spine, shortened long bones, and a left clubfoot. A detailed anatomy ultrasound performed in our center showed a male fetus with an echolucent tubular structure anterior to the spinal canal and separate from the aorta, coursing from the head to the sacrum (Figure 1). Color Doppler did not show any flow through the structure (Figure 2). Hypomineralization of the vertebrae was also noted. The estimated fetal weight was 454 grams, corresponding to the 12th percentile (Hadlock). Head and abdomen measurements were normal while all long bones were small, lagging 3 to 4 weeks in growth. There were no fractures or significant limb bowing. Skull mineralization was normal; there was no frontal bossing but micrognathia was noted. There was no bell shaped chest; heart-to-chest measurement and the chest-to-abdominal circumference were within normal limits. The size and shape of the scapula were normal. The femur length-to-abdominal circumference was low at 0.15 and the femur to foot ratio was low at 0.8. Amniotic fluid volume was normal.

**Figure 3:**
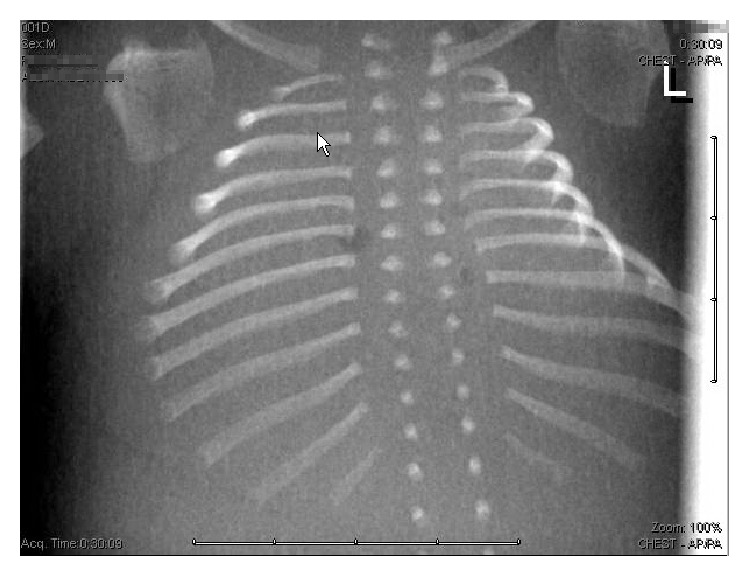
Postmortem radiograph showing complete absence of calcification of the vertebral bodies.

Amniocentesis was performed and a skeletal dysplasia panel using next generation sequencing was utilized and detected a heterozygous mutation in the* COL2A1* gene (c.3598G>A; p.Gly1200Ser). This missense mutation, creating a glycine to serine substitution in the triple helical region of COL2A1, has been previously described as a mutation associated with a diagnosis of hypochondrogenesis [2]. As a femur length-to-abdominal circumference less than 0.16 has been associated with a lethal prognosis, the patient ultimately elected to terminate pregnancy, which was performed in another center [3]. Postmortem skeletal survey radiography showed evidence of poor mineralization of the calvarium and complete absence of ossification of the vertebral bodies (Figure 3), consistent with the diagnosis of hypochondrogenesis.

## 3. Discussion

Hypochondrogenesis is a type II collagenopathy that is caused by a mutation in the* COL2A1* gene located on the long arm of chromosome 12 (locus 12q13.1) [4]. Typical findings on ultrasound include micromelia, poor mineralization of the vertebrae, bowed bones, and equinovarus [5]. Hypochondrogenesis has been associated with a poor prognosis, including hydrops fetalis and early neonatal death from respiratory failure [2]. Although we do not have autopsy confirmation, the tubular structure on ultrasound is consistent with a persistent notochord, being located anterior to the spinal canal and associated with hypomineralization of the vertebrae. The sonographic appearance of the notochord on prenatal imaging has been described by Postma et al. in fetuses with a brachyury mutation and is identical to our findings [6].

The notochord is an embryonic structure found in all chordates. Being the most prominent axial skeletal component, it provides structure to the early embryo allowing normal elongation. The notochord is also involved in creating left-right asymmetry through the expression of specific signals [1]. In higher vertebrates such as the human, it is usually a transient structure that ultimately forms the nucleus pulposus of the intervertebral discs [1]. Interestingly, the notochord is a primitive cartilage. During embryonic development, it expresses genes that encode for SOX9, chondromodulin, and type II, type IX, and type X collagen [7–10]. Most importantly, type II and type X collagen lead it to be replaced by bone therefore forming the vertebrae. Between vertebrae, type X collagen is not expressed allowing the notochord to become the nucleus pulposus of the intervertebral discs [1, 10]. As shown in animal studies, type II collagen is required for the removal of the notochord and normal development of the spine [11]. Therefore, it would be reasonable to infer that deficiency of type II collagen would be associated with a persistent notochord in humans.

To our knowledge, this is the first case of a persistent notochord associated with a* COL2A1* mutation in humans. A persistent notochordal canal has however been described in* COL2A1*-null mice, in which there is an inability to form intervertebral discs [11]. At this time, it is unknown if only a small number of* COL2A1* mutations cause a persistent notochord or if the notochord has been overlooked in other prenatal evaluations given the multitude of other severe features observed in these fetuses.
